# RIG-I is responsible for activation of type I interferon pathway in Seneca Valley virus-infected porcine cells to suppress viral replication

**DOI:** 10.1186/s12985-018-1080-x

**Published:** 2018-10-23

**Authors:** Pengfei Li, Xiangle Zhang, Weijun Cao, Fan Yang, Xiaoli Du, Zhengwang Shi, Miaotao Zhang, Xiangtao Liu, Zixiang Zhu, Haixue Zheng

**Affiliations:** 1 State Key Laboratory of Veterinary Etiological Biology, National Foot and Mouth Diseases Reference Laboratory, Key Laboratory of Animal Virology of Ministry of Agriculture, Lanzhou Veterinary Research Institute, Chinese Academy of Agricultural Sciences, Lanzhou, 730046 China; 2 College of Veterinary Medicine, Northwest A&F University, Yangling, 712100 Shaanxi China

**Keywords:** RIG-I, SVV, Interferon, Immune response, Viral replication

## Abstract

**Background:**

Retinoic acid-inducible gene I (RIG-I) is a key cytosolic receptor of the innate immune system. Seneca valley virus (SVV) is a newly emerging RNA virus that infects pigs causing significant economic losses in pig industry. RIG-I plays different roles during different viruses infections. The role of RIG-I in SVV-infected cells remains unknown. Understanding of the role of RIG-I during SVV infection will help to clarify the infection process of SVV in the infected cells.

**Methods:**

In this study, we generated a RIG-I knockout (KO) porcine kidney PK-15 cell line using the Clustered Regularly Interspaced Short Palindromic Repeats (CRISPR)/CRISPR-associated protein-9 nuclease (Cas9) genome editing tool. The RIG-I gene sequence of RIG-I KO cells were determined by Sanger sequencing method, and the expression of RIG-I protein in the RIG-I KO cells were detected by Western bloting. The activation status of type I interferon pathway in Sendai virus (SeV)- or SVV-infected RIG-I KO cells was investigated by measuring the mRNA expression levels of interferon (IFN)-β and IFN-stimulated genes (ISGs). The replicative state of SVV in the RIG-I KO cells was evaluated by qPCR, Western bloting, TCID_50_ assay and indirect immunofluorescence assay.

**Results:**

Gene editing of RIG-I in PK-15 cells successfully resulted in the destruction of RIG-I expression. RIG-I KO PK-15 cells had a lower expression of IFN-β and ISGs compared with wildtype (WT) PK-15 cells when stimulated by the model RNA virus SeV. The amounts of viral RNA and viral protein as well as viral yields in SVV-infected RIG-I WT and KO cells were determined and compared, which showed that knockout of RIG-I significantly increased SVV replication and propagation. Meanwhile, the expression of IFN-β and ISGs were considerably decreased in RIG-I KO cells compared with that in RIG-I WT cells during SVV infection.

**Conclusion:**

Altogether, this study indicated that RIG-I showed an antiviral role against SVV and was essential for activation of type I IFN signaling during SVV infection. In addition, this study suggested that the CRISPR/Cas9 system can be used as an effective tool to modify cell lines to increase viral yields during SVV vaccine development.

## Background

Clustered regularly interspaced short palindromic repeats (CRISPR) exists widely in bacteria and archaea, which provides acquired immunity and causes interference against foreign bacteriophages invasion [1, 2]. The initial discovery of the CRISPR was in *Escherichia coli* in 1987 [3]. CRISPR loci consists of 21- to 48-base pair (bp) noncontiguous direct repeats separated by non-repetitive sequences called spacers, and flanked by diverse CRISPR-associated genes (*Cas* genes) [4]. *Cas* genes encode an allogenetic and more complex family of proteins that perform similar functions as helicases, nuclease, and RNA-binding proteins [5].

Upon the locus organization and gene conservation, CRISPR has been divided into three main types: type1, type2 and type3 [6]. Among them, the type2 CRISPR system has been used broadly in the field of genome editing in various species and cell types. The type2 CRISPR system includes the Cas9 nuclease, a noncoding trans-activating crRNA (tracerRNA) and a precursor crRNA [7].The crRNA fuses to the tracerRNA guiding the Cas9 protein to bind with the target DNA sequences causing a strand-specific cleavage [8]. A double-stranded break (DSB) is introduced in the chromosome and then the damaged DNA is repaired by non-homologous end joining (NHEJ) pathway. Insertions or deletions (indels) are generated by NHEJ, leading to frameshift mutation or premature stop codons and destruction of gene expression [9]. CRISPR-Cas9 system has a series of advantages such as simple to design, efficient, cheap, and relatively accurate genome editing in host cells comparing with the others genome editing technologies, such as zinc-finger nucleases (ZFNs) and transcription activator-like effector nucleases (TALENs) [10].

Innate immune system recognizes pathogens by pattern recognition receptors (PRRs) including Toll-like receptor (TLRs), RIG-I-like receptor (RLRs), nucleotide oligomerization domain-like receptor (NLRs) and cytosolic DNA sensors [11, 12]. PRRs-mediated antiviral signaling induces the expression of interferons (IFNs), proinflammatory cytokines and IFN-stimulated genes (ISGs) during viral infections [13]. RLRs are cytosolic RNA sensors, consisting of three members: retinoic acid-inducible (RIG-I), melanoma differentiation-associated gene 5 (MDA5), and laboratory of genetics and physiology-2 (LGP2). RIG-I has a DExH/D helicase core flanked by two N-terminal tandem CARDs and a C-terminal domain (CTD) [14, 15]. RIG-I detects a wide variety of RNA viruses, such as influenza A virus, newcastle disease virus (NDV) and Ebola virus [16–18].

RIG-I mainly recognizes free 5′ triphosphate end structures and short dsRNAs; and MDA5 mainly recognizes long dsRNA [12, 19]. Seneca valley virus (SVV) is a newly emerging RNA virus that belongs to the genus *Senecavirus* of family *Picornaviridae*. SVV infects pigs and causes vesicular disease in pigs that presents similar clinical signs with foot-and-mouth disease (a highly contagious and economically devastating viral disease of pigs and ruminants). As a newly emerging virus in pigs, there are many aspects of the virus and the disease that are not yet fully understood, the mechanisms about the sensing, replication and pathogenesis of SVV have not been clarified. Foot-and-mouth disease virus (FMDV) is the causative agent of foot-and-mouth disease, which also belongs to the family *Picornaviridae* [20]. FMDV is mainly sensed by MDA5 but not RIG-I in the infected cells [21]. However, the role of RIG-I in SVV-infected cells remains unknown.

In present study, we developed a porcine RIG-I knockout (KO) cell line using the CRISPR-Cas9 gene-editing technology and evaluated the role of RIG-I in SVV-infected porcine cells. The established RIG-I KO cell line can be used as a good model for investigation of RIG-I-associated immune response in pig cells; and we determined that RIG-I presented an antiviral role against SVV and was essential for activation of type I IFN signaling during SVV infection. These data provide an insight for increase of SVV propagation during vaccine production by knockout of RIG-I from the engineering cell lines.

## Materials and methods

### Cells, virus and reagents

Porcine kidney PK-15 cells were cultured in DMEM/High Glucose supplemented with 10% fetal bovine serum and 1% penicillin-streptomycin. SVV was isolated previously and preserved by our lab [22]. T7 endonuclease 1 (T7E1) was purchased from New England Biolabs (NEB). Mouse polyclonal antibody against RIG-I was purchased from Cell Signaling Technology. Mouse monoclonal antibody against β-actin was purchased from Santa Cruz Biotechnology (Santa Cruz). The rabbit polyclonal antibody against SVV viral structural protein VP2 was prepared by our lab.

### Construction of RIG-I CRISPR-Cas9 plasmids, transfection and single-cell isolation

Three sgRNA sequences were designed by an online CRISPR Design Tool (http://crispr.mit.edu/), targeting the first exon of porcine RIG-I gene (Table 1). The BbsI sites were added in the 5′-terminal of sgRNA sequences. The synthesized sgRNA oligonucleotides were resuspended (100 μM) and mixed, and then were annealed by the following conditions: 95 °C for 5 min and then ramp down from 95 °C to 85 °C at 2 °C/s; then ramp down from 85 °C to 25 °C at 0.1 °C/s. The annealed sgRNA oligonucleotides were cloned into the CRISPR-Cas9 vector pX330 plasmid (Addgene plasmid: 42230) at BbsI site by using T4 Ligase. PK-15 cells were seeded into 12-well plates (3 × 10^5^ cells). The pX330-sgRNA plasmids or pX330 vector plasmids (2 μg) were transfected into PK-15 cells using electrophoretic transfer (Nucleofector™ 2b Device System). The transferred cells were reseeded in 6-well plates at 37 °C. After 72 h post-transfection, the cells were divided into two parts. Genomic DNA was extracted from one part of cells and used for analysis of RIG-I genomic DNA. Another part was cultured continuously forpreservation. After the effectiveness of sgRNA was confirmed by T7 endonuclease cleavage assay, the transfected cells were diluted and cultured to isolate single cell clone by limiting dilution method. The amounts of the transfected cells were counted and serially diluted to a density of 0.5 cell per well. The diluted cells were seeded in 96-well plate for monoclonal cultivation for about 1 week. The cells grown from single-cell clone were selected for genomic analysis. The extracted genomic DNA from these single-cell clones was used as template to amplify the target region sequence and evaluate the knockout consequence. The detection primers (Forward: 5′-AAGTGGTTACACCGCATACA-3′; Reverse: 5′-CACCTCAAACTCCGACAATC-3′) were used to amplify the target region sequence. The PCR products were cloned into pMD18-T vector. Eight plasmids per sample were sequenced to confirm frameshift mutation at allele gene.

**Table 1:** The designed sgRNA sequences targeting porcine RIG-I gene

sgRNA	Forward	Reverse
sgRNA1	**CACCG**ACGCTTTCGGGGACTATGTC	**AAAC**GACATAGTCCCCGAAAGCGT**C**
sgRNA2	**CACCG**CGGCGGAATCTGCACGCTTT	**AAAC**AAAGCGTGCAGATTCCGCCG**C**
sgRNA3	**CACC**GCGGAATCTGCACGCTTTCG	**AAAC**CGAAAGCGTGCAGATTCCGC

### T7 endonuclease 1 (T7E1) cleavage assay

The efficiency of three sgRNA sequences for introduction of indels in RIG-I genomic DNA was compared. The genomic DNA was extracted using the DNA Quick DNA extraction kit (QIAGEN) from the pX330-sgRNA plasmids transfected cells according to the manufacturer’s instructions. A pair of detection primers was used to amplify the genomic region that includes the target site. 200 ng of PCR products were denatured and re-annealed according to following conditions: 95 °C for 5 min and then ramp down from 95 °C to 85 °C at 2 °C/s; then ramp down from 85 °C to 25 °C at 0.1 °C/s. The annealed DNA products were treated with T7 endonuclease l for 15 min at 37 °C. The digested products were then subjected to electrophoresis on 1.5% agarose gel.

### Viral infection

RIG-I KO or wildtype (WT) PK-15 cells were cultured in 12-well plates. The monolayer cells were incubated with SVV at multiplicity of infection (MOI) of 0.1 for 1 h at 37 °C. The supernatant was removed and the cells were maintained with DMEM with 1% FBS as previously described [23].

### TCID_50_ assay

The 50% tissue culture infective dose (TCID_50_) assay was performed as previously described [23]. PK-15 cells were seeded on 96-well cell culture plates. The monolayer cells were inoculated with 100 μl of 10-fold serial dilutions of samples and were tested in eight replicates. The cells were cultured until cytopathic effects were clearly observed. The TCID_50_ values were determined using the Reed-Muench method [24, 25].

### Western bloting analysis

The Western bloting assay was performed as previously described [26]. Briefly, the RIG-I KO or WT PK-15 cells were lysed in the lysis buffer and boiled at 95 °C. The lysates were then subjected to sodium dodecyl sulfate-polyacrylamide gel electrophoresis (SDS-PAGE). The proteins were transferred onto a nitrocellulose membrane (Pall) after running gel and were incubated with appropriate antibodies. The antigen-antibody complex was visualized by Supersignal West Pico Chemiluminescence Substrate purchased from Thermo Scientific.

### Real-time qPCR

Total RNA was extracted from cells using the TRIzol reagent (Invitrogen) and the cDNA was synthesized via reverse transcription using avian myeloblastosis virus reverse transcriptase following the manufacturer’s instructions. The quantitative PCR experiment was performed using the TB Green Premix ExTaq reagents (TaKaRa) on the QuantStudio5 QPCR system (Applied Biosystems). Relative expression of mRNA was normalized to the expression of glyceraldehyde-3-phosphate dehydrogenase (GAPDH) using the 2^−ΔΔCT^ method. All qPCR specific primers are listed in Table 2.

**Table 2:** The qPCR primers used in this study

Gene	Primers(5′ → 3′)
SVV	Forward: AGAATTTGGAAGCCATGCTCT
	Reverse: GAGCCAACATAGARACAGATTGC
GAPDH	Forward: ACATGGCCTCCAAGGAGTAAGA
	Reverse: GATCGAGTTGGGGCTGTGACT
IFN-β	Forward: GCTAACAAGTGCATCCTCCAAA
	Reverse: AGCACATCATAGCTCATGGAAAGA
MXA	Forward: GAGGTGGACCCCGAAGGA
	Reverse: CACCAGATCCGGCTTCGT
GBP1	Forward: AGCACCTTCGTCTACAACAGC
	Reverse: TCAGCCGAGTCCTCAATCC

### Indirect immunofluorescence assay (IFA)

RIG-I KO or WT PK-15 cells were seeded in 12-wells and mock-infected or infected with SVV for 10 h. The cells were fixed with 4% paraformaldehyde for 30 min and then permeabilized with 0.25% Triton X-100 at room temperature for 5 min. After three times washes with phosphate-buffered saline (PBS), the cells were blocked with 5% bovine serum albumin in PBS for 30 min and then incubated with anti-VP2 antibody at 4 °C overnight. The cells were then washed for five times with PBS and incubated with Alexa-Fluor 488 goat anti-rabbit secondary antibody for 30 min, followed by incubation with 4,6-diamidino-2-phenylindole (DAPI) for 5 min. After washing with PBS, the fluorescent images were acquired using Leica Microsystems.

### Statistics and data analysis

All experiments were performed in triplicate and at least three times. Significant differences were calculated using Student’s t test. ^*^P < 0.05 was considered as significant, ^**^P < 0.01 and ^***^P < 0.001 were taken as highly significant.

## Results

### Knockout of RIG-I using CRISPR/Cas 9 system

**Fig. 1:**
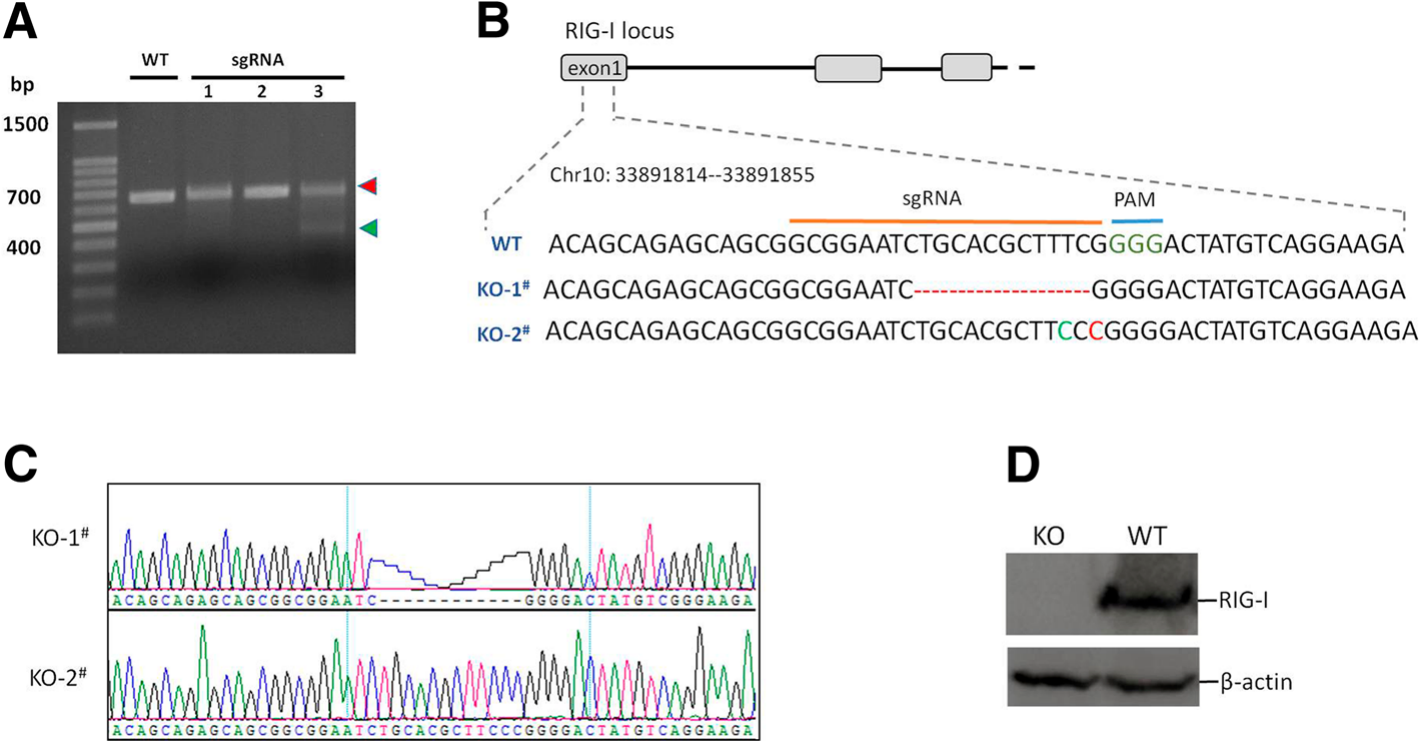
Generation of a RIG-I KO PK-15 cell line by CRISPR/Cas9 system. **a** The electrophoresis results of the T7E1 assay. Red arrow showed PCR amplicons and green arrow indicated the cleaved fragments. **b** The sgRNA3 targeted sequences in the RIG-I gene. GGG (green) was the PAM locus. A bi-allelic modifications were presented in KO-1^#^ and KO-2^#^. The dashed line (red) respresented deletion and highlighted bases indicated indels. **c** Sanger sequencing results showing the indels into the two alleles. **d** Western bloting analysis of RIG-I expression in the RIG-I KO and WT PK-15 cells. β-actin was used to normalize the protein loading quantities

To generate RIG-I KO PK-15 cells, we designed three RIG-I-specific CRISPR-Cas9 sgRNAs targeting the first exon of the porcine RIG-I. The sgRNA was cloned into the pX330 vector using the BbsI terminal sequences. The pX330-sgRNA1, pX330-sgRNA2 and pX330-sgRNA3 plasmids were transfected into PK-15 cells respectively. The T7E1 assay indicated that there were indels introduced into the RIG-I genome resulting from transfection of pX330-sgRNA3 plasmid (Fig. 1a). The PK-15 cells transfected with pX330-sgRNA3 were seeded into 96-wells plates for single-cell screening by the limiting dilution method (0.5 cell each well). The genomic DNA extracted from the single-clone cells were used as the template for amplification of the genomic region that included the target site. The amplified fragments were cloned into pMD-18 T vectors. Eight plasmids for each single-cell clone sample were sequenced to confirm the frame-shifting mutation of both alleles of the established cell line. We successfully acquired one homozygous RIG-I KO cell line with 11 nucleotides deletion in one allele and one nucleotide insertion in another allele of RIG-I genome (Fig. 1b). The Sanger sequencing results indicated the clean sequence peaks were obtained (Fig. 1c).

### RIG-I protein is undetectable in the established KO cell line

To further confirm the silence of RIG-I expression in the screened RIG-I KO cell line, the RIG-I protein expression in the RIG-I WT and KO PK-15 cells were evaluated by Western bloting analysis. The results showed that RIG-I protein was not detected in the RIG-I KO PK-15 cells, however, it can be detected from the RIG-I WT cells (Fig. 1d). These data suggested that the RIG-I KO PK-15 cell line was successfully established.

### Investigation of IFN-β and ISGs expression in RIG-I KO PK15 cells infected with SeV

**Fig. 2:**
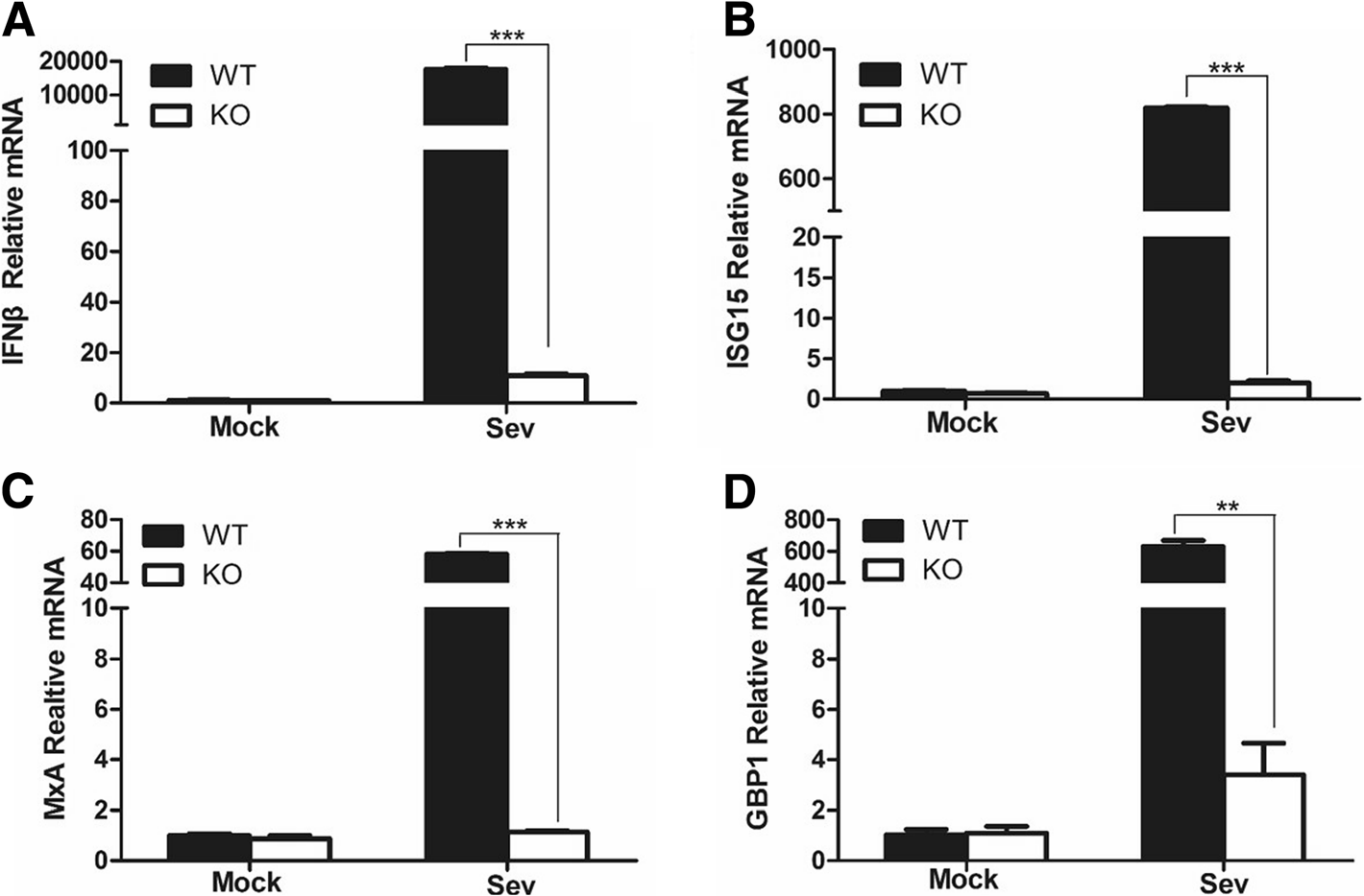
RIG-I KO PK-15 cells showed higher levels of IFN-β and ISGs than that in the WT PK-15 cells upon Sev stimulation. RIG-I KO and WT PK-15 cells were mock-infected or infected with SeV (100 HAU/ml) for 12 h. The expression levels of IFN-β (**a**), ISG15 (**b**), MxA (**c**)), GBP1 (**d**) were evaluated by qPCR

RIG-I acts as a cytosolic receptor that plays a significant role in sensing invading pathogens. RIG-I is implicated in recognition of vesicular stomatitis virus, Sendai virus (SeV), NDV and many RNA viruses. After detecting RNA virus, RIG-I activates the signal transduction of type I IFN signal pathway and induces the expression of IFN-β, proinflammatory cytokines and hundreds of ISGs, suppressing viral replication by various mechanisms. SeV is a model RNA virus routinely used to activates type I IFN pathway signaling in cell culture [27–29]. We analyzed the transcript levels of IFN-β and several ISGs including ISG15, MXA and GBP1 in RIG-I WT and KO cells infected by SeV. SeV infection considerably induced IFN-β and ISGs expression in RIG-I WT PK-15 cells. However, in the RIG-I KO PK-15 cells, SeV infection triggered remarkably weak expression of IFN-β and ISGs (Fig. 2). These results suggested that RIG-I was an important sensor in PK-15 cells to induce the expression of IFN-β and ISGs, and the RIG-I KO PK-15 cells carried a defective type I IFN signal pathway.

### Evaluation of SVV replication in RIG-I KO PK-15 cells

**Fig. 3:**
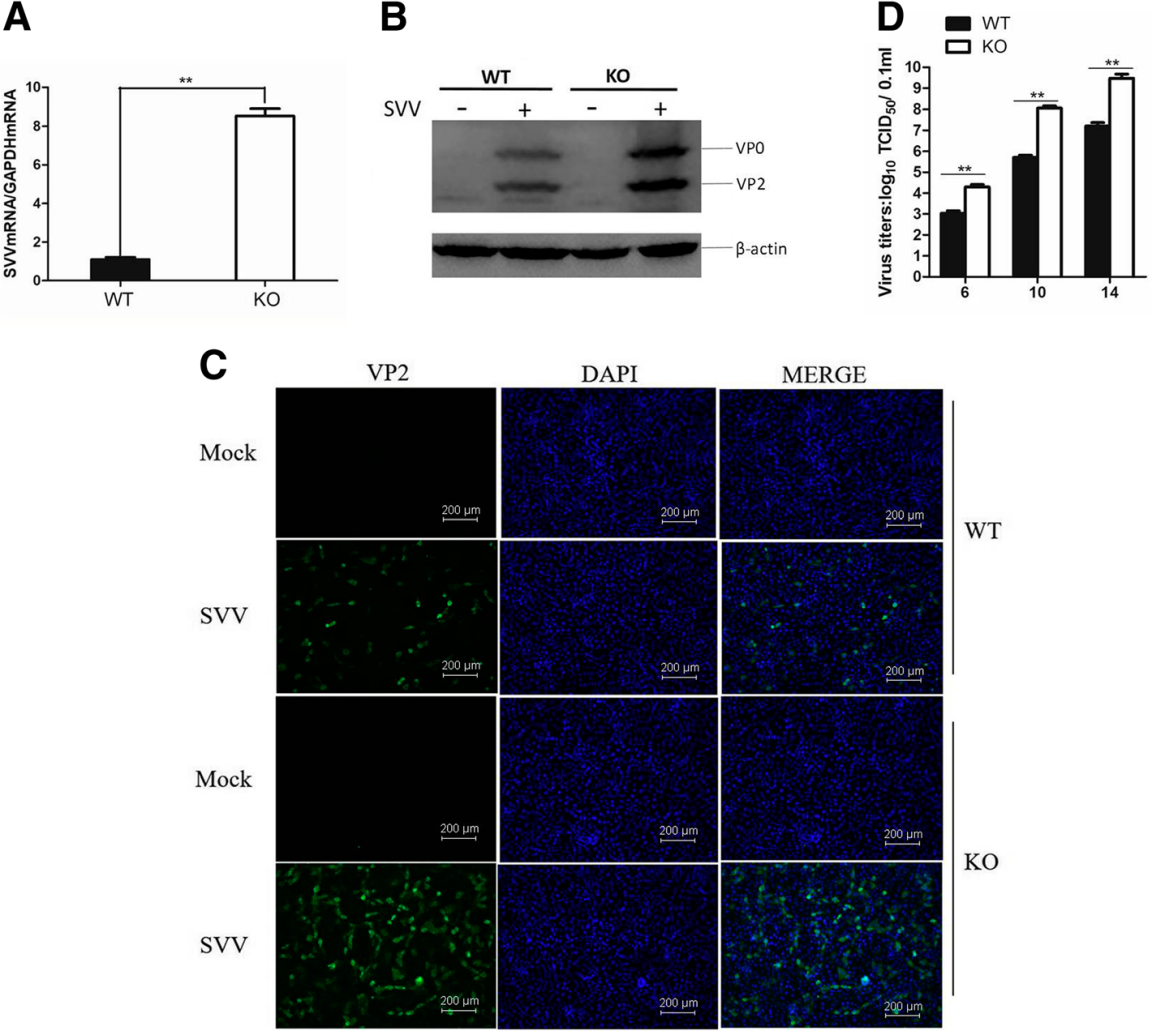
Knockout of RIG-I enhanced SVV replication in PK-15 cells. **a** RIG-I KO and WT PK-15 cells were infected with SVV (MOI of 0.1) respectively, and the cellular samples were collected at 10 hpi. Viral RNA levels were detected by qPCR, (**b**) viral protein levels were detected by western bloting, and (**c**) viral protein levels were detected by IFA. **d** RIG-I KO and WT PK-15 cells were infected with equal amounts of SVV for 6, 10 or 14 h, the viral titers were detected by TCID_50_ assay

As a newly emerging disease, SVV has caused a significant economic loss to pig industry. However, many aspects of the mechanisms about SVV remain unknown. Our previous study has showed that PK-15 cell was permissive for SVV infection [22]. To investigate the potential role of RIG-I in PK-15 cells, we evaluated the replication status of SVV in both RIG-I KO and WT PK-15 cells. The same amount of RIG-I KO and WT PK-15 cells were incubated with equal amount of SVV (0.1 MOI). The viral RNA and viral protein levels were determined at 10 h post infection (hpi). As shown in Fig. 3a and b, both the viral RNA and viral protein levels were higher in RIG-I KO PK-15 cells than that in RIG-I WT PK-15 cells. The expression of viral proteins was also detected by IFA. A considerably higher expression of SVV proteins were observed in RIG-I KO PK-15 cells than that in RIG-I WT PK-15 cells (Fig. 3c). The viral yeilds in SVV-infected RIG-I WT and KO cells at different time points were further measured and compared, which also suggested that SVV replicated more rapidly in the RIG-I KO cells than that in the WT cells (Fig. 3d). These results indicated that RIG-I played an antiviral function during SVV infection in PK-15 cells.

### RIG-I is essential for activation of type I IFN signal pathway during SVV infection in PK-15 cells

**Fig. 4:**
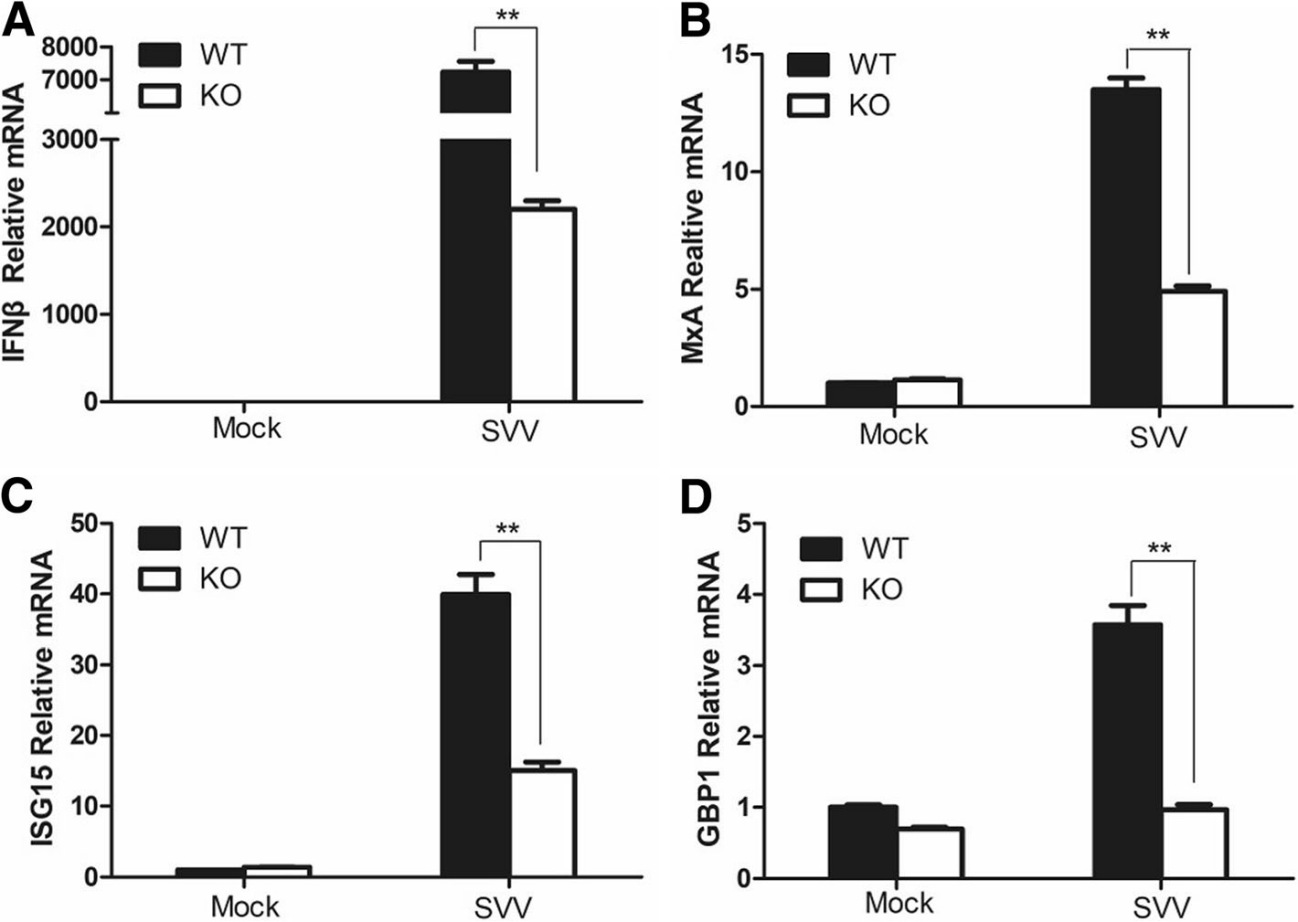
RIG-I was essential for type I IFN signal pathway activation during SVV infection. RIG-I KO and WT PK-15 cells were mock-infected or infected with SVV at an MOI of 0.1 for 10 h. The expression levels of IFN-β (**a**), MxA (**b**), ISG15 (**c**), GBP1 (**d**) were measured by qPCR

Knockout of RIG-I significantly increased SVV replication. To investigate whether RIG-I is involved in type I IFN signal pathway activation during SVV infection, the expression of IFN-β and several ISGs including ISG15, MXA and GBP1 in RIG-I WT and KO cells infected by SVV was detected. SVV infection also triggered high expression of IFN-β and ISGs in RIG-I WT PK-15 cells (Fig. 4), suggesting that SVV infection activated type I IFN signaling. However, the expression of IFN-β and ISGs was significantly decreased in SVV-infected RIG-I KO PK-15 cells. This indicated that SVV infection activated type I IFN signal pathway, and RIG-I was essential for the activation of type I IFN signal pathway to induce IFN-β and ISGs expression. RIG-I might be responsible for viral RNA recognition during SVV infection.

## Discussion

CRISPR/Cas9 system has been widely used in generation of gene modification cell lines or animals. In the transgenic research in pigs, a number of knock-in or knockout porcine cells and pigs have been generated using CRISPR/Cas9 system [30–33]. Such as the critical region of fusion receptor of porcine reproductive and respiratory syndrome virus (PRRSV), CD163 subdomain 5 has been knocked out from porcine immune cells. The knockout monocyte and macrophage cells were completely resistant to PRRSV infection [34]. In present study, we generated an innate immune sensor RIG-I knockout porcine cell line using the CRISPR/Cas9 system. Western bloting analysis showed that RIG-I protein was completely depleted in the established RIG-I KO cell line (Fig. 1). Because the indels were introduced into the first exon of RIG-I gene, we asserted that RIG-I expression had been completely destructed in the RIG-I KO cells.

After sensing of pathogen-associated molecular patterns (PAMPs), RIG-I is activated and interacts with mitochondrial antiviral-signaling protein (MAVS) to induce the production of type I IFN and various cytokines [11, 12]. Type I IFN subsequently activates the janus kinase (JAK) signal transducer and activator of transcription (STAT) pathway to induce numerous ISGs expression [35]. The high expression of cytokines and ISGs plays crucial functions to inhibit virus replication [36]. RIG-I also induces the adaptor molecule STING expression and restricts virus infection [37]. In various human and mouse cells, depletion of RIG-I results in decreased expression of IFNs, proinflammatory cytokines and ISGs during several virus infection [12, 38, 39]. However, few studies have been performed to investigate the situation in the porcine cells. In this study, we established an RIG-I KO PK-15 cell line. Knockout of porcine RIG-I also resulted in the decrease of SeV-induced IFN-β and ISGs expression as compared that in the RIG-I WT cells (Fig. 2). This indicated that RIG-I also performed an import role in innate immune signaling in porcine cells.

SVV was first isolated from a culture contaminant in 2002, and the genome sequence was first confirmed in 2005. SVV is initially used as an oncolytic virus for treatment of cancer [40]. Recently, outbreaks of SVV infection in pigs have emerged and rapidly spread in many countries and caused severe economic loss [22, 41, 42]. We isolated an epidemic SVV strain CH-FJ-2017 (GenBank: KY747510.1) from pigs previously. To investigate the role of RIG-I in SVV-infected cell, we compared viral replication of CH-FJ-2017 and expression of IFN-β and several ISGs between RIG-I KO and WT PK-15 cells. We determined that RIG-I showed an antiviral role against SVV, and found that knockout of RIG-I decreased the expression of IFN-β and several ISGs during SVV infection (Figs. 3 and 4). Gene knockout studies have distinguished the roles of RIG-I and MDA5 in response to RNA viruses. FMDV is mainly sensed by MDA5, however, in the present study, RIG-I is essential for type I IFN pathway activation during SVV infection. This implies that RIG-I might be responsible for sensing of SVV. SVV includes a shorter viral genome (~ 7.2 kb) compared with FMDV genome (~ 8.5 kb), the sensing of two viruses by host cells might be different.

Knockout of RIG-I considerably increased SVV propagation. At present, there are no available commercial vaccines against SVV. Our previous study developed an inactivated vaccine that might be used as a potential vaccine to prevent SVV spread [43]. Here, we showed that establishment of a RIG-I KO cell line using the CRISPR/Cas9 system is a strategy for increasing the viral yields of SVV. This indicates that the CRISPR/Cas9 system can be used as an effective tool to modify cell lines to increase viral yields during SVV vaccines development, and RIG-I gene is a potential target.

## Conclusions

RIG-I plays different roles during different viruses infections. Basing on knockout of RIG-I from porcine cells, we determined that RIG-I is responsible for sensing of SVV and activation of type I interferon pathway in SVV-infected cells. RIG-I played a significant antiviral role against SVV. SVV is a newly emerging RNA virus that infects pigs and causes significant economic losses in pig industry. This study helped to understand the infection process of SVV in the infected cells and also suggested that the CRISPR/Cas9 system can be used as an effective tool to modify cell lines to increase viral yields during SVV vaccine development.

## Abbreviations

CRISPR-Cas9: Clustered Regularly Interspaced Short Palindromic Repeats/CRISPR-associated protein-9 nuclease
INF-β: interferon-β
ISGs: IFN-stimulated genes
KO: knockout
PRRs: pattern recognition receptors
RIG-I: Retinoic acid-inducible gene I
SeV: Sendai virus
SVV: Seneca valley virus
WT: wildtype
